# Characterization and phylogenetic analysis of the complete mitochondrial genome of Mango tilapia (*Sarotherodon galilaeus*: Cichlidae)

**DOI:** 10.1007/s11033-023-08288-6

**Published:** 2023-02-13

**Authors:** Yosur G. Fiteha, M. A. Rashed, R. A. M. Ali, M. Magdy

**Affiliations:** 1 Genetics Department, Faculty of Agriculture, Ain Shams University, Cairo, Egypt; 2 Zoology Department, Faculty of Women for Arts, Science and Education, Ain Shams University, Cairo, Egypt

**Keywords:** *Sarotherodon galilaeus*, Mitochondrial genome, NGS, Phylogeny, Cichlid

## Abstract

**Background:**

*Sarotherodon galilaeus* (Linné, 1758) is a member of the family Cichlidae, which is considered the most important aquaculture freshwater species endemic to Africa and the Middle East. The genetics and molecular biology of this species are rare. This requires more comprehensive mitochondrial genomes-based phylogenetics to enhance understanding of the relationship and delineate this species.

**Methods and results:**

Here, we assembled the complete mitogenome of *S. galilaeus* using Illumina high-throughput sequencing technology. The mango tilapia mitogenome was 16,631 bp in length with an AT composition of 53.4% and 46.4% GC content. It encodes 37 genes comprising two ribosomal RNA genes (rRNAs), 22 transfer RNA genes (tRNAs), and 13 protein-coding genes (PCGs) as well as the D-loop known as the control region. The phylogenetic tree was conducted to provide a relationship within the haplotilapiine lineage based on the maximum likelihood method, and the newly sequenced *S. galilaeus* was clustered with other *Sarotherodon* species.

**Conclusion:**

Our results provide a new perception of the genetic basis of *S*. *galilaeus* species for further research on systematics, evolution, population genetics, and molecular ecology.

## Introduction

The great radiations of African cichlid fishes are the most diverse extant and provide a peculiar and powerful model system in speciation and adaptive radiation research [1]. African cichlids commonly known as Tilapia, are a group of paraphyletic species referred to as haplotilapiine lineage and constitute the most diverse subclades within the cichlids [2]. The cichlid’s diversity was observed in the Great Lakes region of East Africa that evolved over the past 10 million years and represents a peculiar case of fast speciation known among all vertebrates. The great lakes provided a suitable environment for that spectacularly rapid sequence of speciation events that produced one of the most diverse endemic species assemblages, in terms of morphology, behavior, and ecology [3].

*Sarotherodon galilaeus* (Linné, 1758) species of the family Cichlidae, native species to Africa and Northern Africa (Egypt) but is widely cultivated in Europe and America [4]. It possesses a variety of attributes that make it ideal for freshwater aquaculture, as they adapt to a range of environmental conditions, grows rapidly, reproduces easily, and accepts artificial fish feeds. It has a tasty flesh with a mild flavor but is widely accepted as food in many cuisines. Being highly abundant in Egypt, it forms one of the constant supplies of Egyptian tilapiines in the local market [5].

The conserved features of coding content, rapid evolution, accelerated mutation rate, and lack of recombination of the mitochondrial genome have been extensively used as biomarkers for molecular research. Currently, portions of the mitochondrial genome are the frequent markers used to resolve relationships among diversifying zoological taxa and provide information on the distributional boundaries of genetically divergent species [6, 7]. Thus, those markers have been used in areas such as phylogenetic molecular evolution, evolutionary genomics, and population genetics [8].

Recently, few publications have dealt with the morphological diversity and taxonomical delimitation of the *S. galilaeus* species, while the genetics studies and molecular biology of this species are rare. Even though the complete mitochondrial genome of *S. galilaeus* has been reported previously, their result was unclear and uncertain, especially when the reported mitogenome was clustered with *Oreochromis aureus* rather than other Sarotherodon species [9]. Therefore, the present study aimed to resequence and report the complete mitogenome of *S. galilaeus* collected from their natural and original habitat (the Nile River). The mitogenome will provide a base for comparative phylogenetic analysis among other members of Nile tilapiine from the GenBank database to enhance our understanding of the tilapiine biodiversity in the Egyptian freshwaters and expand the genetic resources available for future comparisons among cichlid fishes.

## Materials and methods

### Sample collection and DNA extraction

The fish under study *S. galilaeus* was collected from Manzala Lake, Egypt (31.3306° N, 32.0497° E) during June 2020. The morphological characteristics of the specimen were carefully examined and identified up to the species level, using the standard taxonomic key of Trewavas [10]. Fin tissues were stored in absolute ethanol and transported to the laboratory for further processing.

Total genomic DNA was extracted from fin tissues using GenElute™ Mammalian Genomic DNA Miniprep Kits (Sigma, Germany), according to the manufacturer’s instructions, with a final elution volume of 50 µl. The isolated DNA was tested for quality by 1% gel electrophoresis, visualized under UV light using the Ingenius3 Gel documentation system (Syngene, UK), and the DNA concentrations were determined with Quantus™ Fluorometer (Promega, USA).

### Library construction, mitogenome assembly, and annotation

Illumina paired-end (PE) shotgun libraries were prepared using the standard protocol of the TruSeq library preparation kit (Illumina, San Diego, California, USA) following the manufacturer’s instructions, and sequenced using Illumina HiSeq 4000 platform (Novogene, China) with 350 bp insert size at 11x sequence depth. High-quality clean reads were filtered, and de novo assembly was conducted using the single-contig approach [11]. The assembled mitochondrial genome was annotated using the online tool Geseq with default parameters [12]. tRNAscan-SE 2.0 was used to predict tRNAs [13], through their anticodon sequence and the typical cloverleaf secondary structure. All the coding sequences were confirmed and corrected by translation using Geneious R10 [14]. The online mitochondrial visualization tool OGDRAW [15] was used to draw the graphical map of the complete mitogenome.

### Phylogenetic analysis

The phylogenetic relationships between *S. galilaeus* and other cichlid species retrieved from the NCBI GenBank database were aligned using the MAFFT aligner [16], implemented in Geneious R10. The phylogenetic tree was inferred based on the whole mitogenome using maximum likelihood methods and the tree was computed using FastTree V2 [17], implemented in Geneious R10.

## Results and discussion

### Mitogenome composition and organization

**Fig. 1:**
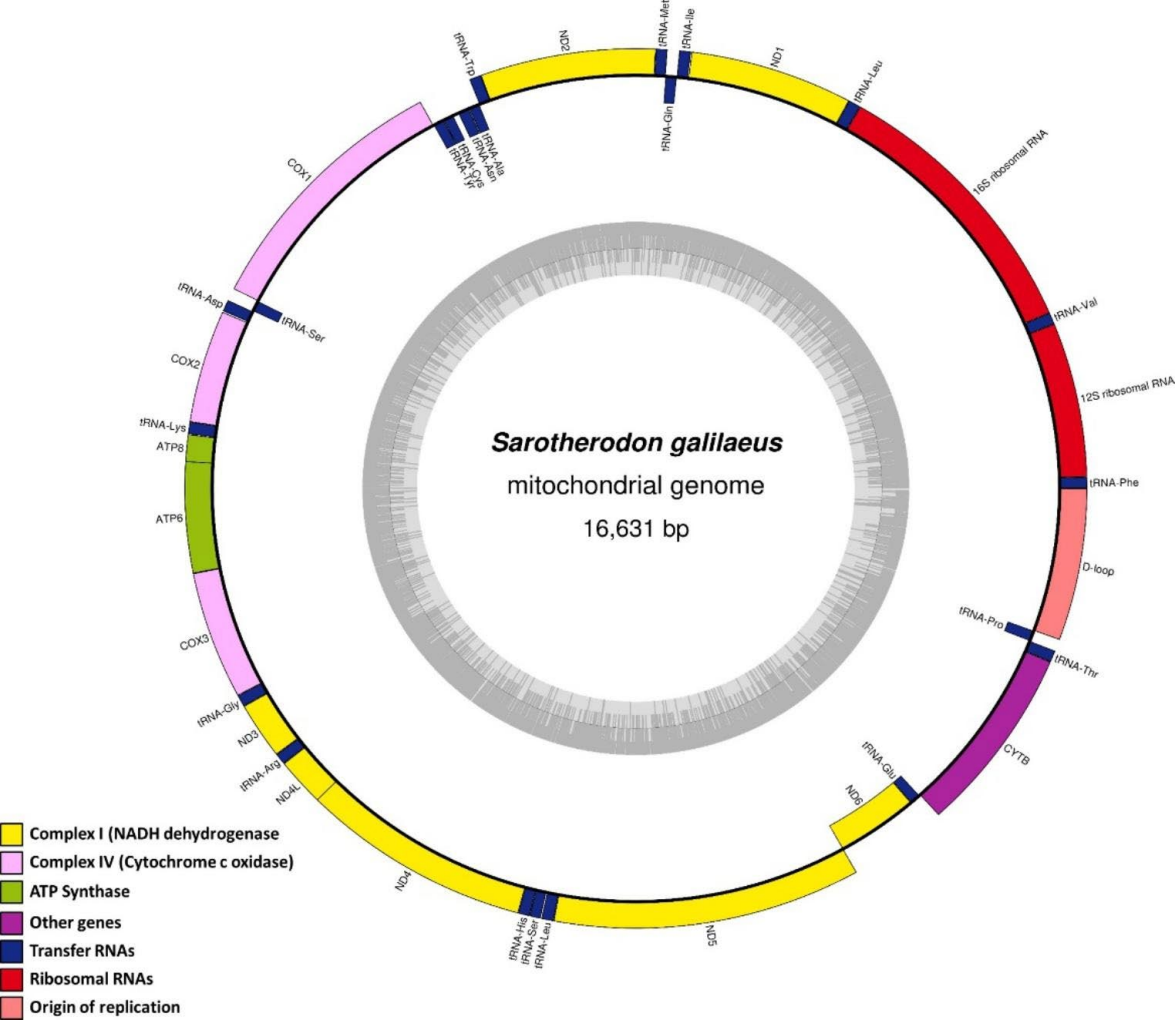
The complete mitogenome map of *S. galilaeus*. The H-strand and L-strand are shown outside and inside. Grey bars inside the circle represent the total GC content, while lighter gray indicates AT content. The size of the mitogenome is 16,631 bp. The annotated map illustrates 13 coding sequences (CDSs), two rRNAs (12 S rRNA and 16 S rRNA), 22 tRNAs, with numerals differentiating each of the two leucine- and serine-specifying tRNAs, and the putative control region (D-loop). Genes belonging to different functional groups are color coded.

As expected, the structure of the newly sequenced *S. galilaeus* is similar to other cichlids belonging to haplotilapiine lineage (e.g., *O. niloticus*, *O. aureus, O. variabilis*, and *Coptodon zillii*) [18, 19], in terms of synteny and genomic features. The sequence was deposited into the GenBank database (Accession number: MW194078). The complete mitochondrial genome of the *S. galilaeus* is 16,631 bp in length, which is a typical circular double-stranded DNA genome (Fig. 1). It contains 13 PCGs, 22 tRNAs, two rRNAs, and a D-loop non-coding region. The basic composition of *S. galilaeus* was found to be A = 27.9%, G = 15.6%, T = 25.5%, and C = 31.0%, and the AT content (53.4%) was higher than the GC content (46.4%) consistent with the patterns observed in other vertebrates (e.g., *Etroplus canarensis*) [20]. The *S. galilaeus* species was observed to exhibit the guanine-rich (H) strand and cytosine-rich (L) strand coding pattern reported for other teleosts. The gene position in each strand has been determined by annotation from databases considering the typical formation and gene composition [21].

### Protein-coding genes

The length of the protein-coding genes (PCG) ranged from 168 to 1839 bp and has a total length of 11,474 bp, and these genes account for 68.99% of the total length of the genes (Table 1; Fig. 1). The gene that has the highest number of base pairs (1839 bp) was recorded for the ND5 coding DNA sequence (CDS), while the lowest (168 bp) was recorded for the ATPase8. The total GC% composition was 47.4 and AT% was 52.6. Twelve of the PCGs were encoded by the heavy strand, while only one (ND6) was encoded by the light strand (Table 1). ATG was the initiation methionine codon used for all PCGS except CO1 which was initiated with GTG, a common finding in other animal mitochondrial DNAs [22]. On the other hand, two stop codons were employed TTA (ATPase6, ATPase8, COI, ND2, ND4L, ND5, and ND6), and TAG (ND1). Incomplete stop codons were detected for CYTB, COX2, COX3, ND3, and ND4, common in the protein-coding genes of the teleost mitochondria [18, 20, 23, 24].

### Ribosomal and transfer RNA genes

The small 12 S rRNA and large 16 S rRNA were identified, recording 945 bp and 1,694 bp, respectively (Table 1), which was within the reported range for vertebrate mitogenomes [18]. Both genes were located near to each other, between trnL^UAA^ and trnF, but separated by trnV. The nucleotides percent compositions of rRNAs were A = 32.3%, C = 27.4%, G = 20.5%, T = 19.9%. Additionally, *S. galilaeus* displayed a higher percentage of AT (52.5%) than GC (47.8%).

Twenty-two tRNAs are detected on the mango tilapia mitogenome counting two for Leucine (L), two for Serine (S), and one for each of the other amino acids (Table 1). Twenty-one tRNA genes showed the classical cloverleaf secondary structure with four domains. One remaining trnS^GCU^ missing the D domain (D-stem and D-loop), a feature commonly observed in metazoan mtDNAs [25]. Fourteen tRNAs were encoded on the H-strand, whereas the remaining tRNAs were encoded on the L-strand (trnQ, trnA, trnN, trnC, trnY, trnS^UGA^, trnE, and trnP; Table 1). All tRNAs varied in size, the lowest was 66 (trnC) and the highest was 74 bp (trnK and trnL^UAA^), while the sum total length was 1553 bp and accounts for 9.33% of the total genome.

### Non-coding region

The non-coding control region known as D-loop was flanked by trnP and trnF genes in the mitochondrial genomes of *S. galilaeus* (Fig. 1). It recorded a 930 bp length, representing 5.5% of the whole genome. This region was especially AT-rich (65.5%) with a composition: A = 32.4%, T = 33.1%, C = 20.8% and G = 13.8%.

### Phylogenetic analysis

**Fig. 2:**
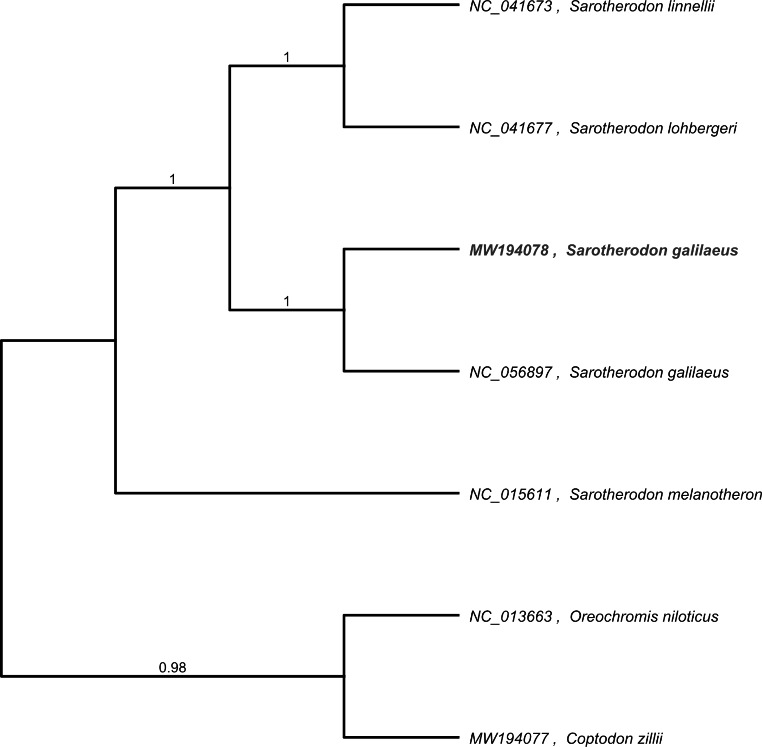
Maximum likelihood phylogenetic tree inferred based on the complete mitogenome of *Sarothrodon galilaeus* and other species within the family Cichlidae, using the *Coptodon zillii* and *Oreochromis niloticus* as an outgroup. Each label includes the GenBank accession number and species name. The number on each node indicates the bootstrap value.

The maximum likelihood inference phylogenetic analysis was performed using the complete mitogenome genomes of *S. galilaeus* and the other six species of haplotilapiine lineage from the Cichlidae family (Fig. 2). *Coptodon zillii* and *Oreochromis niloticus* were chosen as the outgroup for the construction of the phylogenetic tree. Phylogenetic analysis has placed *Sarotherodon* species as sister species within the haplotilapiine lineage. All the species clustered into a single fully supported clade (≥ 98%). The newly sequenced *S. galilaeus* was confirmed as a member of the genus *Sarotherodon*.

In summary, the present results study assembled the mitochondrial genome of *Sarotherodon galilaeus*, an endemic species from southern Egypt. This work provides additional molecular information that can be applied to improve cichlid species identification and help to develop additional markers for population studies and evolutionary analysis.

**Table 1:** Characteristics and features of the mitochondrial genome of *Sarothrodon galilaeus*, including genes, their type, position, length, anticodons for tRNA genes, start and stop codons for PCGs, and strand on which they are encoded

Gene name	Type	Position		Length	Anticodon	Codons		Direction
		From	To			Start	Stop	
trnF	tRNA	1	69	69	GAA	-	-	H
12 S	rRNA	70	1014	945	-	-	-	H
trnV	tRNA	1015	1086	72	UAC	-	-	H
16 S	rRNA	1087	2780	1694	-	-	-	H
trnL	tRNA	2781	2854	74	UAA			H
ND1	CDS	2855	3829	975	-	ATG	TAG	H
trnI	tRNA	3833	3902	70	GAU	-	-	H
trnQ	tRNA	3902	3972	71	UGG	-	-	L
trnM	tRNA	3972	4040	69	CAU	-	-	H
ND2	CDS	4041	5087	1047	-	ATG	TAA	H
trnW	tRNA	5087	5158	72	UCA	-	-	H
trnA	tRNA	5160	5228	69	UGC	-	-	L
trnN	tRNA	5230	5302	73	GUU	-	-	L
trnC	tRNA	5338	5403	66	GCA	-	-	L
trnY	tRNA	5404	5473	70	GUA	-	-	L
COX1	CDS	5475	7070	1596	-	GTG	TAA	H
trnS	tRNA	7071	7141	71	UGA	-	-	L
trnD	tRNA	7145	7217	73	GUC	-	-	H
COX2	CDS	7223	7913	691	-	ATG	T(AA)	H
trnK	tRNA	7914	7987	74	UUU	-	-	H
ATP8	CDS	7989	8156	168	-	ATG	TAA	H
ATP6	CDS	8147	8830	684	-	ATG	TAA	H
COX3	CDS	8830	9613	784	-	ATG	TA(A)	H
trnG	tRNA	9614	9685	72	UCC	-	-	H
ND3	CDS	9686	10,034	349	-	ATG	TAG	H
trnR	tRNA	10,035	10,103	69	UCG	-	-	H
ND4L	CDS	10,104	10,400	297	-	ATG	TAA	H
ND4	CDS	10,394	11,774	1381	-	ATG	T(AA)	H
trnH	tRNA	11,775	11,843	69	GUG	-	-	H
trnS^GCU^	tRNA	11,844	11,910	67	GCU	-	-	H
trnL	tRNA	11,915	11,987	73	UAG	-	-	H
ND5	CDS	11,988	13,826	1839	-	ATG	TAA	H
ND6	CDS	13,823	14,344	522	-	ATG	TAA	L
trnE	tRNA	14,345	14,413	69	UUC	-	-	L
CYTB	CDS	14,418	15,558	1141	-	ATG	T(AA)	H
trnT	tRNA	15,559	15,630	72	UGU	-	-	H
trnP	tRNA	15,631	15,700	70	UGG	-	-	L
D-loop	D-loop	15,701	16,631	931	-	-	-	H

## Data Availability

The genome sequence data that support the findings of this study are openly available in GenBank of NCBI under the accession number MW194078.
